# Development of a Swine Whole Eye Transplant Ex Vivo Perfusion Protocol

**DOI:** 10.1111/aor.70118

**Published:** 2026-03-19

**Authors:** Haïzam Oubari, Lucile Cabanel, Yanis Berkane, Ali Mojallal, Mark A. Randolph, Basak Uygun, Korkut Uygun, Curtis L. Cetrulo, Alexandre G. Lellouch

**Affiliations:** ^1^ Center for Engineering in Medicine and Surgery Massachusetts General Hospital, Harvard Medical School Boston Massachusetts USA; ^2^ Shriners Children's Boston Boston Massachusetts USA; ^3^ Plastic Surgery Research Laboratory, Department of Plastic and Reconstructive Surgery Massachusetts General Hospital, Harvard Medical School Boston Massachusetts USA; ^4^ Department of Plastic, Reconstructive and Aesthetic Surgery Hospices Civils de Lyon Lyon France; ^5^ Department of Plastic, Reconstructive and Aesthetic Surgery and SITI Laboratory UMR1236, INSERM, CHU de Rennes, University of Rennes Rennes France; ^6^ Division of Plastic Surgery Research Vascularized Composite Allotransplantation Program Development, Cedars‐Sinai Hospital Los Angeles California USA

## Abstract

**Background:**

The first human Whole Eye Transplant (WET) has sparked new hope for patients who have lost vision due to major ophthalmic injury, but significant challenges remain to be addressed to achieve vision restoration. The eyeball, especially its neural components, undergoes nearly immediate degeneration unless adequately preserved. Oxygenated subnormothermic machine perfusion (SNMP) has emerged as a promising alternative to static cold storage (SCS), offering potential benefits for graft preservation and reconditioning.

**Methods:**

WETs were procured from adult swine following a mean *164.7* ± *6.1 min* of warm ischemia and perfused ex vivo using a Steen+ solution administered at room temperature for 18 h. Perfusion parameters, weight gain, and metabolic markers were recorded throughout the perfusion. Histological analysis and an atropine mydriasis test were performed to assess WET integrity and function.

**Results:**

Following an initial phase of metabolic clearance and weight loss, perfusion parameters stabilized, and final weight gain (*t* = 18 h) remained below 10%. Histological evaluation confirmed tissue preservation, and iris function was restored upon atropine administration.

**Conclusions:**

This study represents the first application of SNMP to a whole eye composite transplant model, demonstrating promising outcomes in this large animal model and supporting SNMP as a potential alternative to SCS for WET.

## Graphical abstract

This study establishes the first subnormothermic machine perfusion protocol for whole eye transplants in a swine model, enabling up to 18 h of preservation with stable metabolic function, limited edema, and recovery of mydriasis.

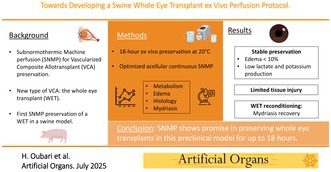

## 1 Introduction

Vascularized composite allotransplants (VCAs) can offer unparalleled outcomes [1, 2], and the recent first whole eye transplant (WET) has sparked new hope for patients suffering from eyeball‐related vision loss by demonstrating the technical feasibility of the procedure, although sight was not restored [3, 4]. Strikingly, functional imaging demonstrated cortical activation in response to light stimulation, and electroretinography (ERG) indicated a blunted but recognizable photoreceptor response to light [5]. The extreme sensitivity of the retina and optic nerve to ischemia is a major concern to be addressed as a priority, as these structures undergo irreversible degeneration within minutes of warm (and cold) ischemia time (WIT) unless proper oxygenation and pH levels are maintained [6]. Thus, WET presents unique challenges within the VCA field; in addition to achieving optic nerve reconnection, preventing ischemic injury to the graft will be essential to ultimately restore vision.

Machine perfusion (MP) is well‐established in solid organ transplantation as a method to prevent ischemia–reperfusion injury [7, 8, 9]. More specifically, subnormothermic machine perfusion (SNMP) has demonstrated superior outcomes compared to static cold storage (SCS) in various models [10, 11, 12] and its potential for graft reconditioning after WIT [13, 14]. SNMP supports acellular perfusate solutions, greatly facilitating clinical settings and minimizing blood‐related risks such as cross‐reactivity, infection, and allo‐immunization. Our group has successfully translated SNMP with modified Steen solutions across animal models ranging from rodents [15, 16] to swine [17, 18, 19] and nonhuman primates [20, 21]. We have developed an SNMP protocol specifically optimized for VCA and applied it to a swine Whole Eye Transplant model.

## 2 Methods

### 2.1 WETs Procurement

Institutional approval for this study was obtained from the local Institutional Animal Care and Use Committee (protocol #2024N000205). All animal work followed the Animal Research: Reporting of In Vivo Experiments guidelines and was in accordance with the US Army Animal Care and Use Review Office recommendations (ACURO). All WETs were procured from swine undergoing terminal organ procurement procedures, in accordance with the “3‐R” principles [22]. The grafts were procured following euthanasia and below a 3‐h warm ischemia limit. Animals weighing 30–50 kg were euthanized by exsanguination after receiving 100 IU/kg of heparin. The left WETs were used for experiments, and the contralateral grafts served as controls. Contralateral eyes were exposed to the same duration of warm ischemia as the perfused WETs but did not undergo ex vivo preservation or machine perfusion; they were directly sampled after warm ischemia for histological analysis and iridomotor function testing. This standardized warm ischemia period, inherent to the exempt tissue protocol design, provided a consistent baseline for evaluating the effect of machine perfusion on graft recovery while partially reflecting donation after circulatory death (DCD) conditions. The WET procurement was based on an established model that was tailored to our study requirements [23, 24] and consisted of the intraorbital content, including the eyeball, oculomotor muscles, and palpebra, pedicled on the external carotid artery system prolonged up until the common carotid artery and jugular vein system through the connection between the external and internal carotid systems (Figure S1).

### 2.2 Subnormothermic Machine Perfusion

**FIGURE 1:**
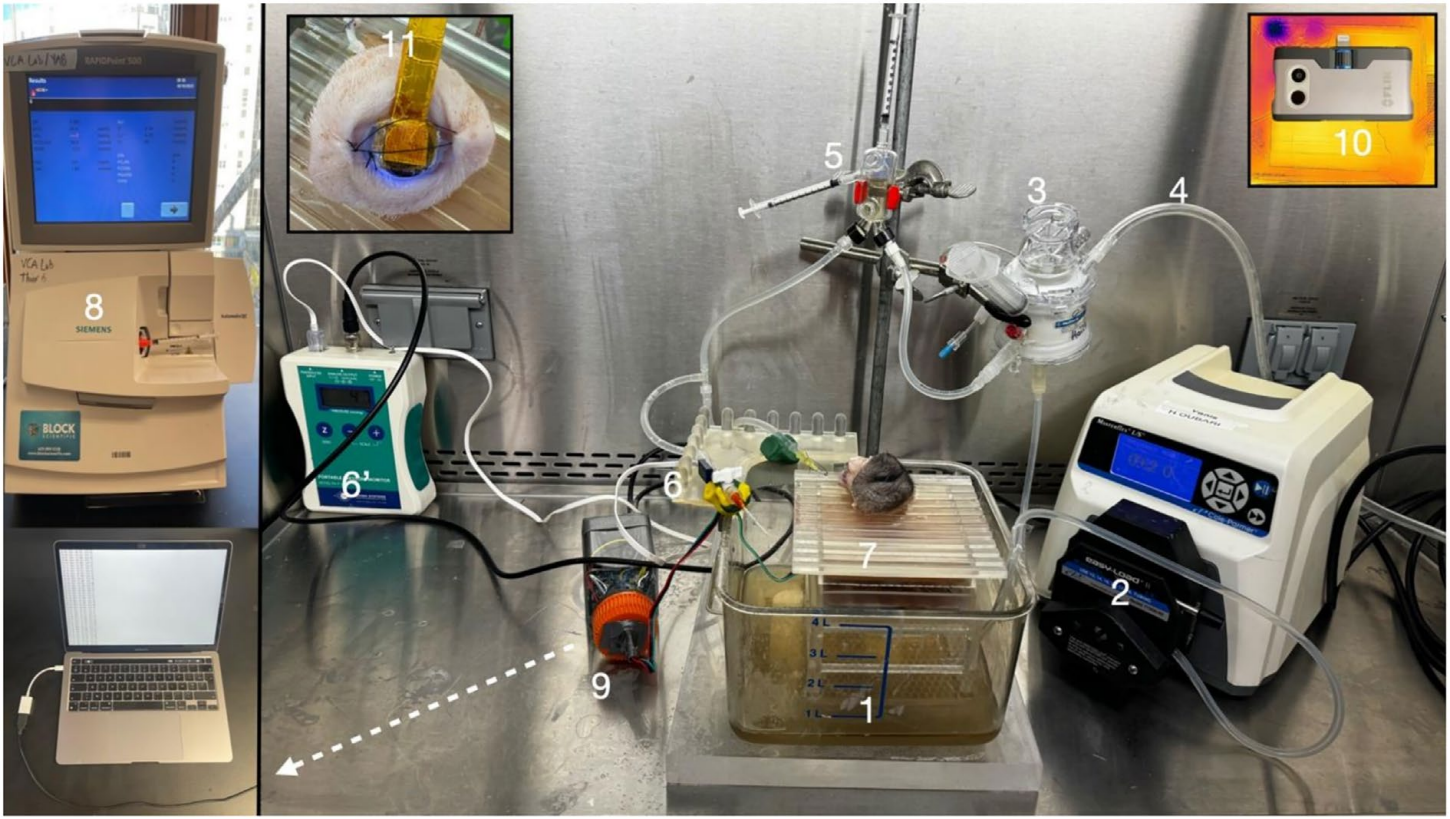
Ex Vivo subnormothermic machine perfusion setup. The perfusion solution (1) is pumped (2) through the oxygenator (3) and aerated with a carbogen mixture (4) (5% CO_2_ and 95% oxygen). The solution then goes through the bubble trap (5) and the pressure is measured (6 and 6′) at WET height. The WET is placed on top of a 3D‐printed scale (7). Inflow is sampled from the stopcock, upstream from the arterial inflow. Outflow samples are taken directly from the vein. Samples are analyzed with a Rapidpoint500 system (8). A microcontroller (9) sends continuous pressure and weight measurements to a computer. Temperature is monitored at each time point with a FlirOne smartphone‐based thermal camera (10) and is kept in the sub‐normothermic range (19°C–21°C) without intervention. A tissue oximetry device was adapted to fit the cornea for real‐time oxygen diffusion monitoring in the eyeball (11). [Color figure can be viewed at wileyonlinelibrary.com]

The perfusion system, described in Figure 1, operates as a semi‐closed recirculating system [13, 25, 26, 27]. The characteristics of our Steen solution optimized for VCAs have already been described. Briefly, a deionized water base was supplemented with ions, albumin, glucose, and steroids. Broad‐spectrum antibiotics (vancomycin and piperacillin–tazobactam) and sodium bicarbonate (8.4%) were added with a targeted pH of 7.4 (Table S1). The perfusion solution was aerated with a carbogen mixture (5% CO_2_ and 95% oxygen). In total, 1.5L of Steen+ solution [28] was used per graft, and the perfusions lasted for 18 h at room temperature (19°C–21°C) with timepoint measurements performed every hour during the first 6 h, then every 3 h. Subnormothermic perfusion is generally defined in the solid organ machine perfusion literature as perfusion performed at temperatures above hypothermic conditions and below normothermia, typically described as above 20°C [11]. In the present study, perfusion was performed at 19°C–21°C, which lies at the lower boundary of subnormothermic conditions. This temperature range was selected to balance metabolic support and edema control while maintaining practical feasibility, as it corresponds closely to ambient room temperature in a laboratory setting and has previously been used in VCA experimentation [29, 30]. Formulas for metabolic and dynamic calculations have already been reported [21]. Briefly, before connecting the WET, the system pressure without the WET was measured at different flow rates (*P*_w/o_). During perfusion, pressures in the WET were observed (*P*_w/_). The vascular pressure (*P*_vasc_) was calculated as:

(1) $$P_{\text{vasc}}=P_{w}/-P_{w/o}$$

The vascular resistance (*R*_vasc_) was calculated as:

(2) $$R_{\text{vasc}}=P_{\text{vasc}/Q}$$

With *Q*, the flow rate.

Lactate and potassium release (Rel) were calculated as follows:

(3) $$\mathrm{Rel}=\left(\left[\text{vein}\right]-\left[\mathrm{art}\right]/W\right)\times 1000\times Q$$

with [vein] and [art] being, respectively, the outflow and inflow concentrations (mmol/l).

Oxygen consumption (O_2cons_) was calculated as the difference between arterial and venous oxygen content and corrected for flow and weight (Equation 1). The following formula was used for calculations:

(4) $$O_{\text{2cons}}=0.0314\times Q^{*}\;\left({\mathrm{pO}}_{2}\;\mathrm{art}–{\mathrm{pO}}_{2}\;\text{vein}\right)/W$$

where 0.0314 corresponds to Henry's constant in water at 20°C at 1 atm, pO2 art‐vein is the difference in partial oxygen pressure between artery inflow and venous outflow (mm Hg), *Q* is the arterial inflow rate (mL/min), and weight (W) is measured in grams and resulting oxygen consumption is measured in mL/min/g. For perfusate analysis, samples were taken from the inflow (corresponding to the carotid artery) and venous outflow (jugular vein). Glucose was included in the perfusate as an energy substrate, but glucose consumption is not reported in this study; metabolic calculations were limited to lactate and potassium release and oxygen consumption.

### 2.3 Structural and Functional Assessments

All major components of the WETs were biopsied after 18 h of ex vivo preservation, fixed in formalin, paraffin‐embedded, sectioned, and stained with hematoxylin and eosin (H&E). Slides were analyzed in light microscopy with a 100× magnification and were evaluated by a blinded, experienced pathologist. Mydriatic function was assessed by injecting 1 mL of atropine into the arterial inflow after 18 h of SNMP in the perfused group and after 3 h of WIT in controls.

### 2.4 Statistical Analysis

Data were recorded in Excel (Microsoft, Redmond, WA, USA), and statistical analyses were performed using Prism (v.10.1.1, GraphPad Software, La Jolla, CA, USA). Continuous variables are presented as mean ± standard error of the mean (SEM). The alpha risk was set at 5% and all tests were two‐tailed. Given the longitudinal nature of the perfusion experiments, temporal trends were analyzed using linear regression. Perfusion time was divided into an early recovery phase (0–6 h) and a late preservation phase (6–18 h). For each phase, regression slopes were calculated and tested against zero to assess the presence of significant temporal changes. Associations between perfusion parameters were explored using Spearman rank correlation coefficients.

## 3 Results

### 3.1 WET Model

**FIGURE 2:**
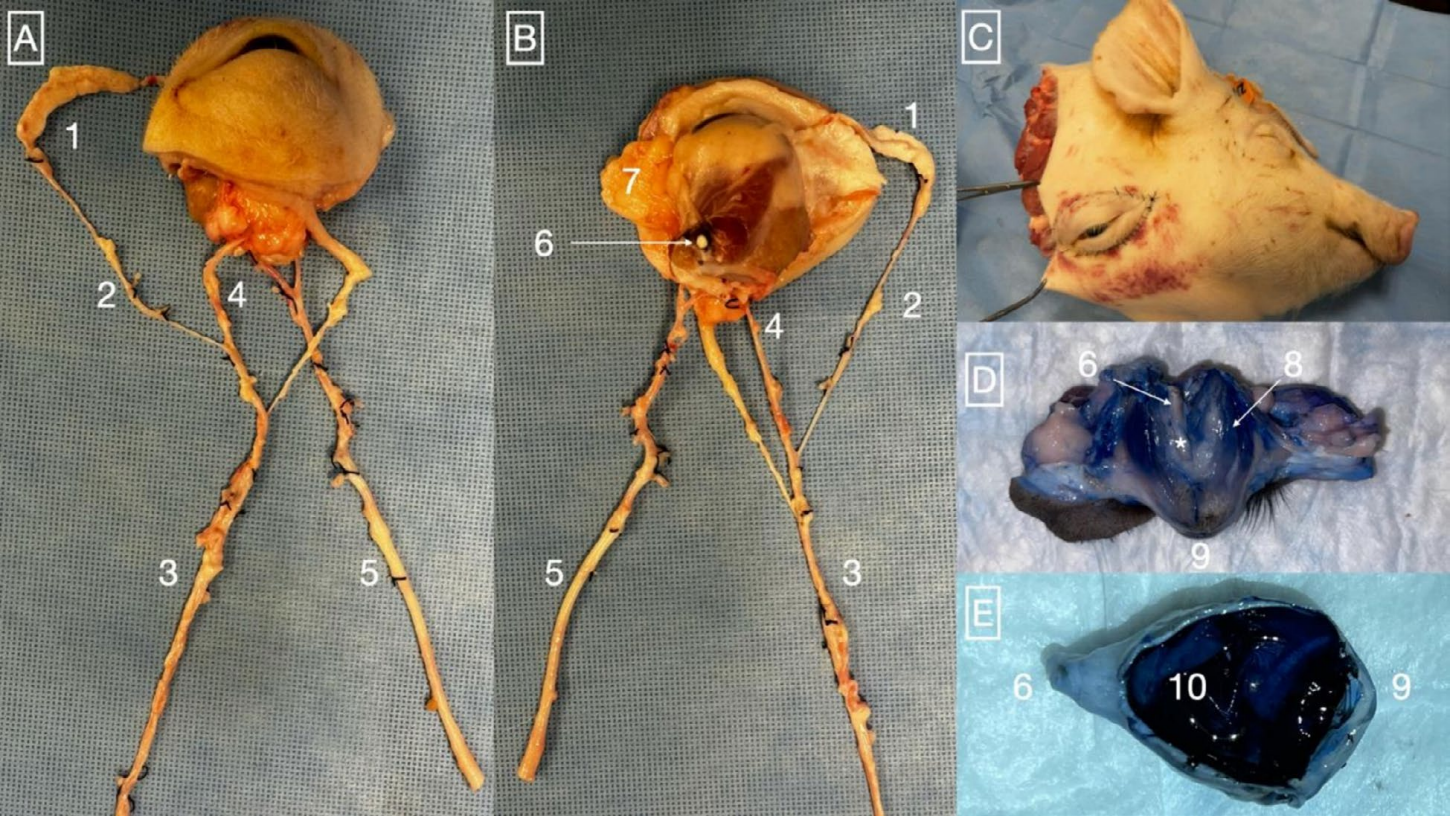
WET anatomical model. (A) Anterior view of the WET; (B) Posterior view of the WET; (C) Superior open view of the WET after blue dye injection in the arterial pedicle; (D) Superior open view of the eyeball following blue dye injection; (E) Lateral view of the WET being reperfused with whole blood after 6 h of SNMP pretest. In this dissection, the frontal vein (1) was preserved and dissected along with the facial vein (2) and the external jugular vein (3). Note the communicating branch (4) between the ophthalmic vein and the facial vein in Figure 1B. The arterial pedicle can be dissected from the ophthalmic artery up to the external and common carotid arteries in the neck (5). In the posterior view of the WET (B), the optic nerve (6) and the lacrimal gland (7) are visible. To prevent leaks during perfusion, the ophthalmic branches connecting to the internal carotid system adjacent to the optic nerve were carefully ligated. To assess the proper vascularization of the WET through the external carotid pedicle, blue dye injections were performed, confirming that all graft components (C) and eyeball segments (D) were properly stained. Note the staining of the posterior ciliary arteries (*) along the optic nerve (6). A subsequent perfusion pretest also confirmed proper recoloration of the entire graft following whole‐blood reperfusion after 3 h of WIT and 6 h of SNMP (E). [Color figure can be viewed at wileyonlinelibrary.com]

Five preliminary dissections were conducted to ascertain the anatomy of the WET grafts and learn the model. No intracranial dissection was required, and the entire orbital content was consistently procured with two arterial branches originating from the external carotid artery and entering the graft at its posterior aspect. The venous system consisted of the ophthalmic veins, which tended to exit the WET more anteriorly than the arterial branches. Additionally, at the anteromedial aspect of the eyeball, a consistent vein was found to join the frontal venous sinus within the frontal bone, before forming the frontal vein and subsequently joining the facial vein more anteriorly. A consistent communicating branch between the frontal vein and the ophthalmic vein was identified, passing below the ascending branch of the mandible. To optimize procurement time for subsequent perfusion experiments, we therefore chose to sacrifice the facial vein as its dissection proved to be tedious (Figure 2A,B). A replantation simulation was also conducted, proving the feasibility of heterotopic cervical replantation of the WET with end‐to‐side vascular anastomoses on the carotid and jugular vessels (Figure 2C). Finally, to ensure proper vascularization of the graft through angiography, arterial injection of blue dye stained all aspects of the WET, confirming complete vascularization (Figure 2D,E).

### 3.2 Ex Vivo Perfusion

**FIGURE 3:**
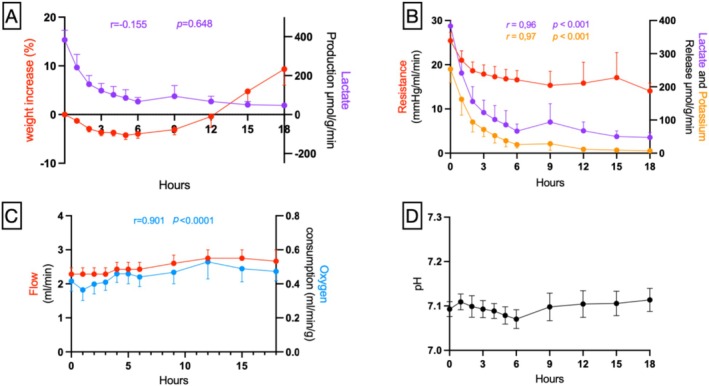
Key metabolic and dynamic parameters during machine perfusion and their correlations. A lack of correlation between lactate levels (a marker of hypoxia) and edema suggests the absence of compartment syndrome (A). Conversely, the correlation between lactate, potassium release, and vascular resistance corroborates the distinction of an initial recovery phase from a subsequent preservation phase (B). Oxygen consumption remained proportional to perfusion flow rate (C) and relatively stable throughout perfusion, as did pH levels (D). [Color figure can be viewed at wileyonlinelibrary.com]

Seven continuous SNMP perfusions were performed. The average WIT was 164.7 ± 6.1 min before perfusion, and the mean initial weight was 62.7 ± 2.4 g. The flow rate was kept stable for all experiments, ranging between 2 and 3 mL/min, resulting in a mean vascular pressure of 57.6 ± 4.0 mmHg at the beginning of the perfusion and progressively decreasing until the end of the experiment, reaching a mean value of 36.7 ± 4.2 mmHg at the end of the perfusion. Linear regression analyses revealed two distinct perfusion phases (Figure S2). The first phase, observed during the initial 6 h, demonstrated significant negative slopes for lactate release, potassium release, and vascular pressure, considered as the *recovery phase*, which coincided with progressive capillary dilation marked by decreasing vascular resistance. The second phase, spanning the subsequent 12 h of perfusion and referred to as the preservation phase, was characterized by the absence of significant temporal trends, with regression slopes not significantly different from zero, indicating stabilization of all parameters (Figure 3). The mean weight intake after 18 h of SNMP was 9.3% ± 3.3%. The pH levels in the perfusate were maintained stable throughout the entire perfusion without the need to add bicarbonate to the solution.

### 3.3 Structural and Functional Assessments

**FIGURE 4:**
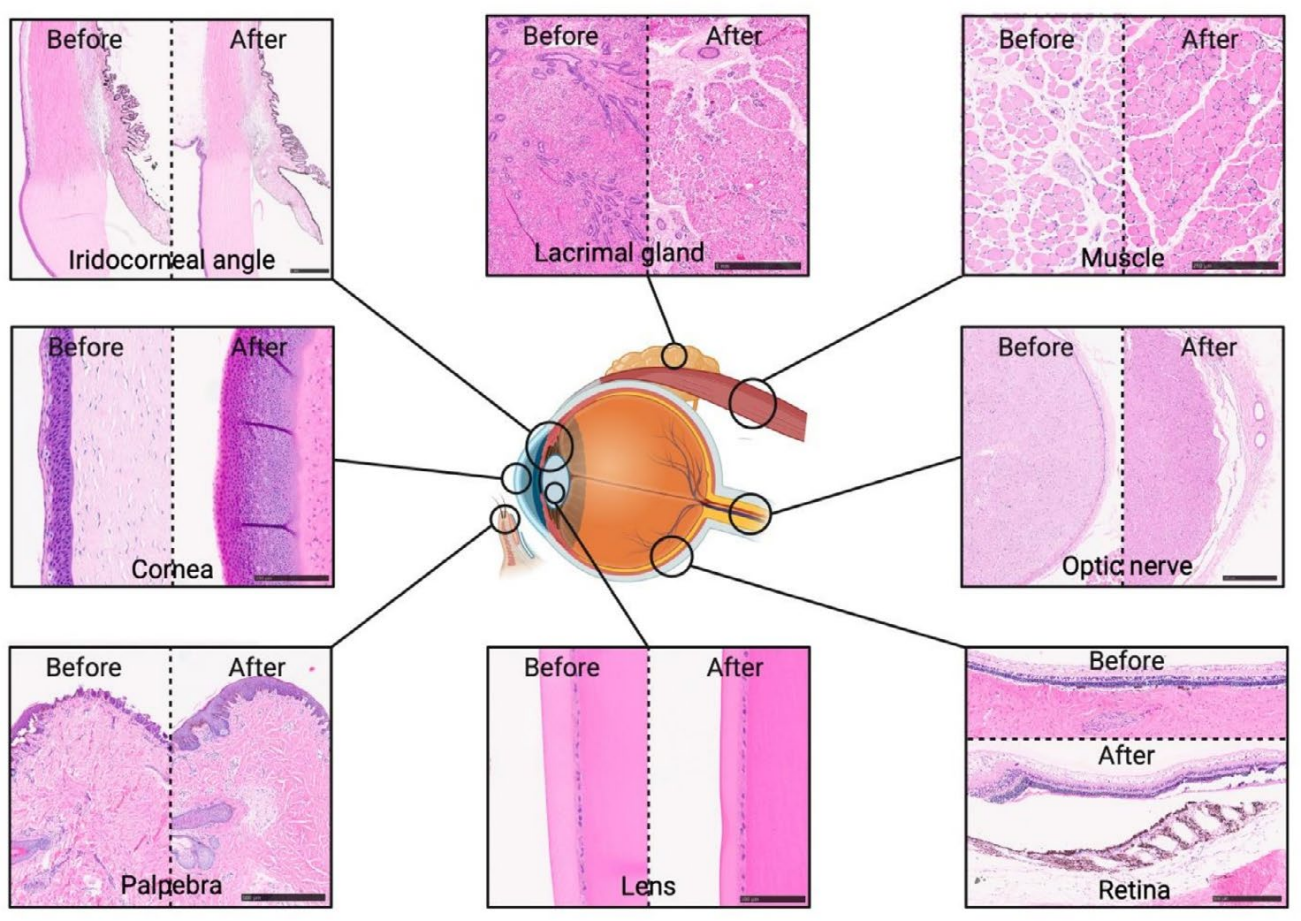
Representative H&E histological analysis of control samples (“before”) and perfused WETs (“after”) across different graft compartments. Most structures, including the cornea, lens, iris, oculomotor muscles, lacrimal gland, and optic nerve, exhibited preserved architecture. The palpebra and conjunctiva showed more pronounced edema. The retina remained relatively well preserved, with tissue damage primarily attributable to biopsy procurement. [Color figure can be viewed at wileyonlinelibrary.com]

Histological analysis demonstrated preservation of overall tissue architecture across sampled WET compartments after 18 h of SNMP. In particular, oculomotor muscle samples exhibited preserved myofiber organization, with intact fascicular architecture, identifiable nuclei, no evidence of major myocyte injury or degeneration, and only limited interstitial edema. The iridocorneal angle remained patent, without gross collapse or architectural distortion. Optic nerve sections showed identifiable axonal bundles and no overt signs of tissue fragmentation or cavitation. Interstitial edema was minimal in most compartments, with the exception of the palpebra and conjunctiva. The lacrimal gland maintained its lobular organization without gross acinar disruption, and both the cornea and lens demonstrated preserved overall architecture, with maintained cellular layering of the corneal epithelium and intact lens structure on H&E. Tissue morphology remained comparable to that observed in the control group (Figure 4).

Atropine administration had no effect in control grafts subjected to 3 h of WIT, but induced mydriasis in all tested WETs following 18 h of SNMP, suggesting perfusion‐induced recovery (Video S1).

## 4 Discussion

More than two decades after the first clinical cases, vascularized composite allotransplantation continues to face significant immunological and ex vivo preservation challenges, limiting its widespread availability [31]. These challenges are even more pronounced in the case of whole‐eye transplantation, where it is particularly crucial to prevent ischemic injury [32, 33, 34] to the eye before transplantation, as the retina can sustain irreversible functional loss within minutes if proper oxygenation and pH levels are not maintained [6].

This study introduces the first protocol for the ex vivo preservation of a new type of VCA, the whole eye transplants (WETs), using subnormothermic machine perfusion (SNMP) in a circulatory death porcine model. Porcine anatomy offers unique advantages, particularly due to the absence of a lateral orbital wall, facilitating straightforward procurement through the external carotid and jugular vessels without intracranial dissection. This makes this model highly suitable for WET preservation and transplantation studies, both orthotopic and heterotopic [23, 24, 35]. The experimental setting mimics a donor in circulatory death, aligning with the “3‐R” principles of refinement, reduction, and replacement [36, 37], potentially opening prospects for expanding the donor pool to heart‐dead donors. This model presents obvious limits to the interpretation of WET viability assessment, since retinal and nervous tissues were exposed to an average of 164 min of warm ischemia. However, this feature enabled proper assessment of functional and post‐ischemia recovery through SNMP. It seems necessary to evaluate WET SNMP in a living‐donor procurement setting in future studies.

Through this preliminary study, we demonstrated that the perfusion parameter during the 18‐h experiments suggests the feasibility of extended WET perfusion, with steady and stable outflow and metabolic profiles. In addition to introducing the longest WET preservation protocol so far, we propose defining two distinct phases: first, a recovery phase that can last up to 6 h and allows for metabolic washout and equilibration of the graft after the WIT. This recovery phase is followed by a preservation phase where metabolic and dynamic markers remain stable, indicating the absence of major ischemia or compartment syndrome. Phase‐specific regression analyses provide quantitative support for this recovery–preservation paradigm. The steep negative slopes observed during the early phase are consistent with rapid clearance of ischemia‐associated metabolites and progressive capillary recruitment, whereas the near‐zero slopes during the later phase indicate a biologically meaningful stabilization rather than a gradual decline in graft condition. Notably, the concordant temporal behavior of lactate clearance, potassium release, and vascular pressure suggests coordinated metabolic and hemodynamic recovery, supporting the interpretation that these parameters reflect interconnected aspects of graft physiology rather than independent readouts. Similar relationships have been described in compartment‐syndrome–sensitive grafts, such as the non‐human primate forearm model, in which lactate and potassium levels strongly correlate with tissue weight gain and vascular resistance, acting as early biological markers of tissue injury [21]. It is important to note that, across transplantation and machine perfusion fields, no universal consensus currently exists regarding single reliable viability biomarkers applicable across organ systems. Accordingly, and consistent with the VCA perfusion literature, we used a combination of metabolic and hemodynamic markers to approximate real‐time graft preservation state during perfusion rather than relying on a single viability parameter. This perfusion approach, in our view, promotes smooth capillary dilation and is more respectful of the capillary structure, allowing for up to 24 h of subnormothermic machine perfusion across various models without causing excessive edema. The total amount of edema was limited and didn't reach the 10% mark that has been observed to increase tissue damage in other VCA models [38]. Most of the edema tended to appear in the conjunctiva and palpebra, while the other tissues showed limited swelling. This observation aligns with the report on the first human eyeball transplant. In this case, performed by Pr. Rodriguez, the edema, although impressive, resolved without causing any ophthalmological issues, and it is to be expected that this will be the case for future transplants performed either in the animal model or in humans. In specifically addressing soft tissue edema, our SNMP protocol, based on a Steen+ solution, loaded with 150 g/L of albumin, demonstrated optimal preservation against interstitial edema, which remains the main limitation in prolonged VCA ex vivo perfusion [39]. While the total amount of edema remained below this threshold, it is important to acknowledge that lower degrees of weight gain may still be biologically relevant. In the ex vivo normothermic limb perfusion model reported by Meyers et al., increases in muscle injury scores as well as elevations in potassium and lactate were observed at approximately 5% weight gain, and lactate and potassium did not consistently correlate with injury severity in that setting. These differences likely reflect model‐ and platform‐specific factors, including tissue composition, compartment sensitivity, perfusion temperature, and perfusate composition. Accordingly, in the present study, weight gain was interpreted as a sensitive early indicator of evolving graft stress rather than a definitive threshold for injury, and stability below 10% should be viewed as supportive but not sufficient evidence of preserved graft viability.

Importantly, the choice of an 18‐h perfusion duration is experimentally pertinent in the context of whole‐eye transplantation and ex vivo studies, as shorter preservation periods (e.g., 6–10 h) can be insufficient to clearly differentiate dynamic perfusion‐based strategies from static cold storage. From a clinical perspective, such a preservation window is also highly relevant, as it would enable long‐distance organ transport and flexible surgical scheduling, both of which are critical considerations for the clinical implementation of whole‐eye transplantation.

The histological findings of this study should be interpreted in the context of an early‐phase, feasibility‐driven evaluation of acellular subnormothermic machine perfusion for whole‐eye transplantation. Preservation of gross tissue architecture across multiple WET compartments suggests that SNMP can maintain basic structural integrity during prolonged ex vivo perfusion. However, these observations provide only a rough estimate of tissue integrity and do not permit conclusions regarding cellular viability, functional preservation, or retinal and optic nerve physiology. More detailed characterization will require targeted histological staining, ultrastructural analyses, posterior segment functional assessments, and ultimately replantation‐based validation, which constitute the focus of ongoing and future studies [6, 40, 41, 42]. Importantly, weight gain during ex vivo perfusion should be interpreted as an early and sensitive indicator of evolving graft injury rather than a binary marker of tissue preservation, as molecular and inflammatory alterations may occur at low levels of edema well before overt histological changes become apparent, particularly in the context of subsequent ischemia–reperfusion injury [43]; accordingly, more comprehensive assessments including transcriptomic, cytokine, and other inflammatory analyses will be required to fully characterize graft viability under SNMP conditions.

This first application of MP in the WET model has potentially major implications for future advances. Together with the development of zero‐ischemia procurement and transplantation strategies, optimized ex vivo perfusion currently represents the only realistic pathway to preserving retinal and optic nerve integrity and, ultimately, to enabling functional vision recovery after WET for the treatment of blindness [4, 5]. Although a major limitation of this first study is the absence of direct posterior segment assessment, due to regulatory and experimental constraints inherent to this pilot work, the recovery of mydriasis provides a strong indicator that the graft can be at least partially reconditioned [14]. In this context, subnormothermic machine perfusion provides a critical platform not only for preservation but also for active graft optimization [30]. In this feasibility study, recovery of a mydriatic response following SNMP should be interpreted as a marker of perfusion‐induced tissue‐level functional preservation or reconditioning of the anterior segment, while more sensitive assessments of posterior chamber and neural functional recovery remain to be investigated in future studies [6]. Prolonged and stable ex vivo preservation opens avenues for graft reengineering aimed at reducing immunogenicity, for metabolic or pharmacologic preconditioning, and for extending preservation durations to support optimized tolerance‐induction protocols [44, 45, 46]. If longer preservation can be achieved, this approach may further enable improved donor–recipient matching, procedural planning, or allow recipient preparation before receiving the graft in a context of a tolerance induction protocol. Beyond transplantation, whole‐eye ex vivo perfusion also establishes a unique multipurpose platform to study ocular compartments in isolation, offering opportunities for diagnostic assessment, therapeutic testing, and fundamental investigation of retinal, vascular, and neural physiology under controlled conditions. Together, these perspectives position ex vivo whole‐eye perfusion not only as a preservation strategy but as an enabling technology for vision‐restorative transplantation and advanced ocular research.

## Author Contributions

H.O. conceived the study, designed the experimental protocol, performed experiments, analyzed the data, and drafted the manuscript. L.C. and Y.B. contributed to experimental work, data collection, and manuscript revision. A.M. contributed to conceptual input and critical revision of the manuscript. M.A.R., B.U., contributed to the development of the perfusion platform, methodological design, and critical revision of the manuscript. K.U., C.L.C., and A.G.L. supervised the project, contributed to study design and data interpretation, and critically revised the manuscript. All authors reviewed and approved the final manuscript.

## Funding

This work was supported by the National Institutes of Health, R01AR082825, R01EB028782. U.S. Department of Defense, RTRP RT240044. Shriners Hospitals for Children, 84308. Advanced Research Projects Agency for Health, FY25.1065.001.

## Conflicts of Interest

Some authors declare competing interests. Drs. Uygun, Lellouch and Cetrulo have patent applications relevant to this study. Dr. Uygun has a financial interest in and serves on the Scientific Advisory Board for Sylvatica Biotech Inc., a company focused on developing high‐subzero organ preservation technology. Korkut Uygun has a financial interest in and serves on the Scientific Advisory Board for Sylvatica Biotech Inc., a company focused on developing high‐subzero organ preservation technology. Competing interests for MGH investigators are managed by the MGB in accordance with their conflicts of interest policies. All the remaining authors declare no conflict of interest.

## Supporting information


**Table S1:** Main perfusate compounds and concentrations in the Steen+ solution.
**Figure S1:** Venous and arterial anatomy enabling whole‐eye transplant procurement in the swine model. Dissection view illustrating the venous connections between the ophthalmic vein, frontal vein, and facial vein, forming a direct communication between the deep orbital venous system and the superficial facial venous network. The facial vein courses superficial to the mandible before draining into the external jugular vein, allowing reliable venous outflow and procurement of the whole‐eye transplant based on the external jugular system. The arterial supply is provided via the external carotid artery, with clear visualization of the ophthalmic artery and associated anastomotic branches. The arterial pedicle can be lengthened up to the common carotid artery after ligating the internal carotid artery. This vascular configuration underlies the feasibility and reproducibility of whole‐eye transplant procurement and ex vivo perfusion in the swine model.
**Figure S2:** Distinct recovery and preservation phases during subnormothermic machine perfusion of whole eye transplants. Linear regression analyses of key metabolic and hemodynamic parameters over time demonstrate two distinct phases during ex vivo perfusion. During the recovery phase (0–6 h, yellow shading), lactate release (purple), potassium release (orange), and vascular pressure (red) showed significant negative slopes, consistent with metabolic washout and progressive capillary recruitment. In contrast, during the preservation phase (6–18 h, blue shading), linear regression slopes were not significantly different from zero for all variables, indicating metabolic and hemodynamic stabilization. Slopes (S) and corresponding *p*‐values are reported on the graph.

**Video S1:** Mydriasis was restored following atropine administration in WET grafts perfused for 18 after 3 h of warm ischemia. In contrast, no pupillary response was observed in control eyes exposed to 3 h of warm ischemia without subsequent perfusion.

## Data Availability

The data that support the findings of this study are available from the corresponding author upon reasonable request.
