# Supplementary figures and images for: Development of a Swine Whole Eye Transplant Ex Vivo Perfusion Protocol
Source: Artif Organs. 2026 Mar 19;50(5):764–72. doi: 10.1111/aor.70118 (PMC13206370; doi:10.1111/aor.70118)

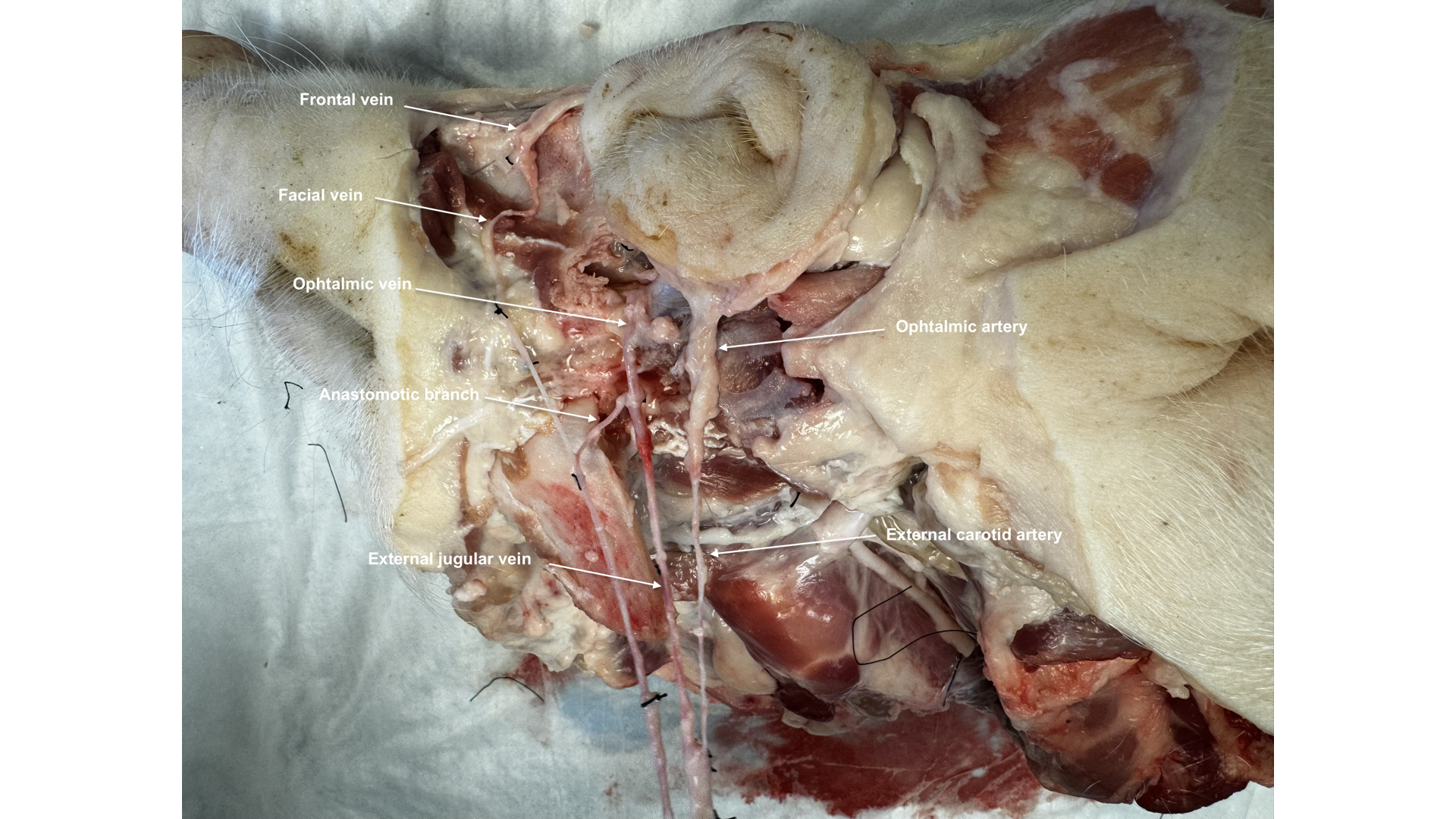

Supplement: Supplementary file 1 — Table S1: Main perfusate compounds and concentrations in the Steen+ solution. Figure S1: Venous and arterial anatomy enabling whole‐eye transplant procurement in the swine model. Dissection view illustrating the venous connections between the ophthalmic vein, frontal vein, and facial vein, forming a direct communication between the deep orbital venous system and the superficial facial venous network. The facial vein courses superficial to the mandible before draining into the external jugular vein, allowing reliable venous outflow and procurement of the whole‐eye transplant based on the external jugular system. The arterial supply is provided via the external carotid artery, with clear visualization of the ophthalmic artery and associated anastomotic branches. The arterial pedicle can be lengthened up to the common carotid artery after ligating the internal carotid artery. This vascular configuration underlies the feasibility and reproducibility of whole‐eye transplant procurement and ex vivo perfusion in the swine model. Figure S2: Distinct recovery and preservation phases during subnormothermic machine perfusion of whole eye transplants. Linear regression analyses of key metabolic and hemodynamic parameters over time demonstrate two distinct phases during ex vivo perfusion. During the recovery phase (0–6 h, yellow shading), lactate release (purple), potassium release (orange), and vascular pressure (red) showed significant negative slopes, consistent with metabolic washout and progressive capillary recruitment. In contrast, during the preservation phase (6–18 h, blue shading), linear regression slopes were not significantly different from zero for all variables, indicating metabolic and hemodynamic stabilization. Slopes (S) and corresponding p‐values are reported on the graph. [file AOR-50-764-s001.zip › Supplemental Figure.1.jpeg]

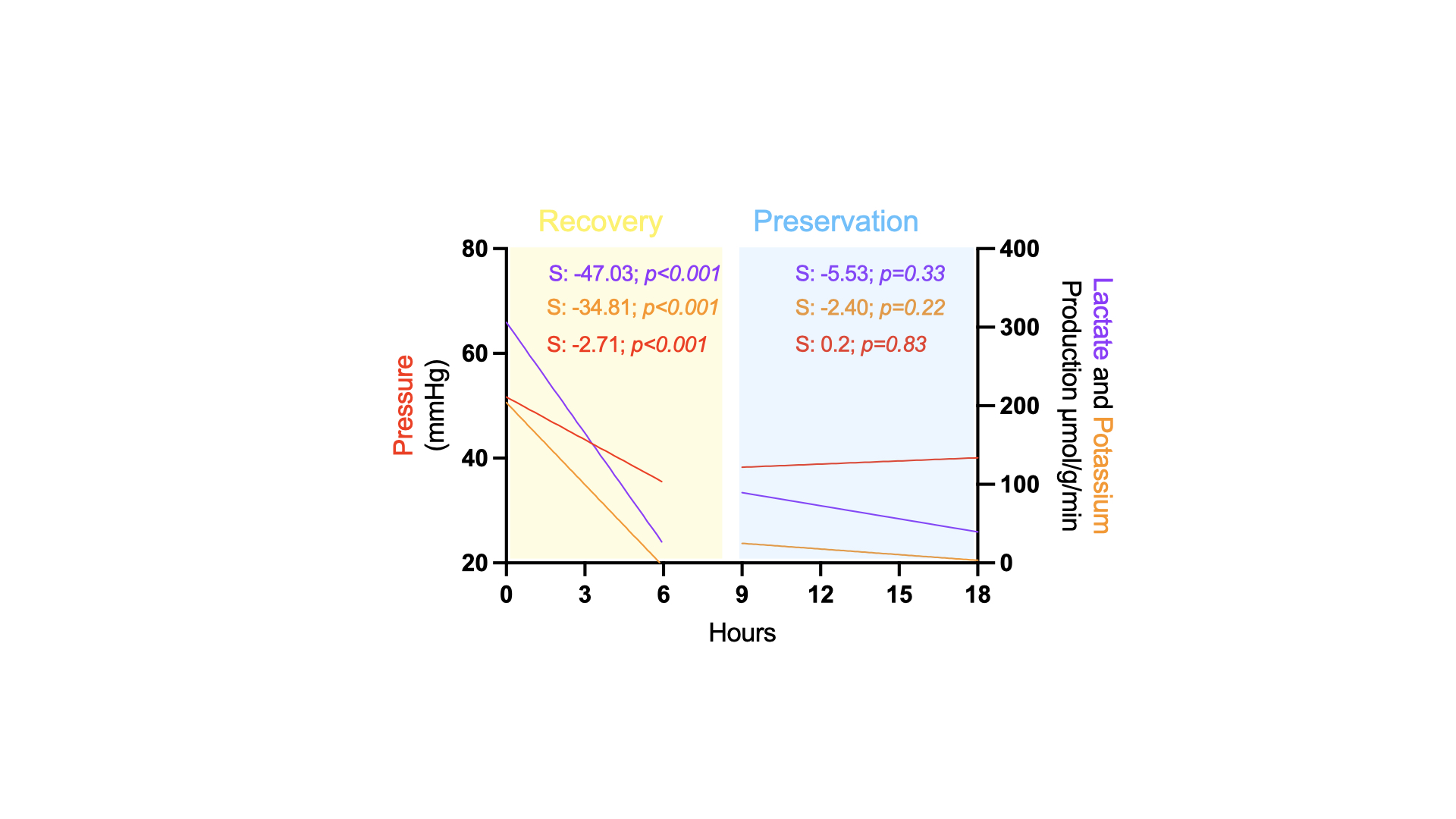

Supplement: Supplementary file 1 — Table S1: Main perfusate compounds and concentrations in the Steen+ solution. Figure S1: Venous and arterial anatomy enabling whole‐eye transplant procurement in the swine model. Dissection view illustrating the venous connections between the ophthalmic vein, frontal vein, and facial vein, forming a direct communication between the deep orbital venous system and the superficial facial venous network. The facial vein courses superficial to the mandible before draining into the external jugular vein, allowing reliable venous outflow and procurement of the whole‐eye transplant based on the external jugular system. The arterial supply is provided via the external carotid artery, with clear visualization of the ophthalmic artery and associated anastomotic branches. The arterial pedicle can be lengthened up to the common carotid artery after ligating the internal carotid artery. This vascular configuration underlies the feasibility and reproducibility of whole‐eye transplant procurement and ex vivo perfusion in the swine model. Figure S2: Distinct recovery and preservation phases during subnormothermic machine perfusion of whole eye transplants. Linear regression analyses of key metabolic and hemodynamic parameters over time demonstrate two distinct phases during ex vivo perfusion. During the recovery phase (0–6 h, yellow shading), lactate release (purple), potassium release (orange), and vascular pressure (red) showed significant negative slopes, consistent with metabolic washout and progressive capillary recruitment. In contrast, during the preservation phase (6–18 h, blue shading), linear regression slopes were not significantly different from zero for all variables, indicating metabolic and hemodynamic stabilization. Slopes (S) and corresponding p‐values are reported on the graph. [file AOR-50-764-s001.zip › Supplemental Figure.2.jpeg]

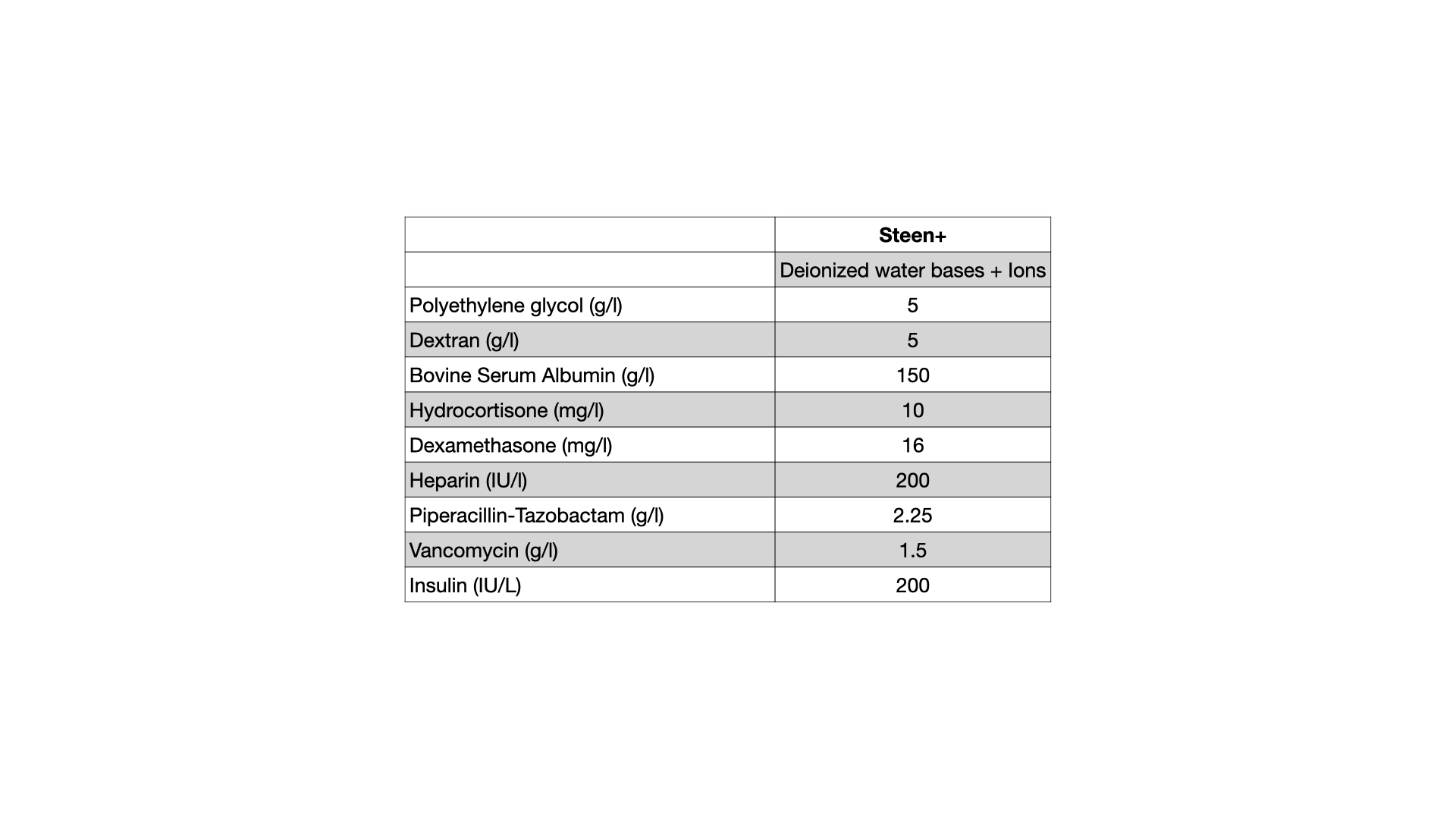

Supplement: Supplementary file 1 — Table S1: Main perfusate compounds and concentrations in the Steen+ solution. Figure S1: Venous and arterial anatomy enabling whole‐eye transplant procurement in the swine model. Dissection view illustrating the venous connections between the ophthalmic vein, frontal vein, and facial vein, forming a direct communication between the deep orbital venous system and the superficial facial venous network. The facial vein courses superficial to the mandible before draining into the external jugular vein, allowing reliable venous outflow and procurement of the whole‐eye transplant based on the external jugular system. The arterial supply is provided via the external carotid artery, with clear visualization of the ophthalmic artery and associated anastomotic branches. The arterial pedicle can be lengthened up to the common carotid artery after ligating the internal carotid artery. This vascular configuration underlies the feasibility and reproducibility of whole‐eye transplant procurement and ex vivo perfusion in the swine model. Figure S2: Distinct recovery and preservation phases during subnormothermic machine perfusion of whole eye transplants. Linear regression analyses of key metabolic and hemodynamic parameters over time demonstrate two distinct phases during ex vivo perfusion. During the recovery phase (0–6 h, yellow shading), lactate release (purple), potassium release (orange), and vascular pressure (red) showed significant negative slopes, consistent with metabolic washout and progressive capillary recruitment. In contrast, during the preservation phase (6–18 h, blue shading), linear regression slopes were not significantly different from zero for all variables, indicating metabolic and hemodynamic stabilization. Slopes (S) and corresponding p‐values are reported on the graph. [file AOR-50-764-s001.zip › Supplemental FTable 1.jpeg]
